# Radiological Imaging Evaluation of the Failing Total Hip Replacement

**DOI:** 10.3389/fsurg.2019.00035

**Published:** 2019-06-18

**Authors:** Nida Mushtaq, Kendrick To, Chris Gooding, Wasim Khan

**Affiliations:** ^1^Department of Trauma and Orthopaedics, Walsall Manor Hospital, Walsall, United Kingdom; ^2^Division of Trauma and Orthopaedic Surgery, Addenbrooke's Hospital, University of Cambridge, Cambridge, United Kingdom

**Keywords:** imaging, hip replacement, complications, computed tomography, nuclear medicine, magnetic resonance imaging

## Abstract

Total hip replacements (THR) have been performed in the UK from the 1960s and since then we have seen surgical techniques, the design of implants, and imaging modalities rapidly develop. This paper will aim to review the different complications and imaging appearance which help to evaluate each problem. As for all investigations for bone and joints, a radiograph is the first imaging to be performed for any patient with a THR and can detect a majority of complications. CT is relatively low-cost, simple to perform and easily available making it an excellent tool to supplement radiographs when trying to evaluate a hip prosthesis. Single photon emission computed tomography with CT (SPECT-CT) is an emerging modality which has shown to combine the sensitivity that bone scintigraphy offers with the high specificity of CT. SPECT imaging also has the advantage of showing the bone's metabolic activity and is less prone to metal artifact than Magnetic resonance imaging (MRI). MRI has evolved to become an important diagnostic tool for the evaluation of THR in the post-operative period. Optimized pulse sequences and metal artifact reduction techniques have made MRI a useful tool in diagnosis of soft tissue abnormalities and is particularly useful in identifying adverse local tissue reactions in metal on metal implants. CT and MRI are accurate in identifying the diagnosis of most causes of THR complications except infection. Research confirms that leukocyte-marrow scintigraphy is the modality of choice for accurately diagnosing prosthetic joint infection and reassures us of its superiority over other nuclear medicine imaging. However, due to the limited availability and increased costs when performing leukocyte-marrow scintigraphy, CT and SPECT-CT would be a more preferred option when suspecting prosthesis infection. Ultrasound (US) has a limited role in the assessment of most THR complications but can be useful to identify peri-prosthetic fluid collections and the presence of soft tissue sinus tracts. Being aware of the imaging modalities that are available to orthopedic surgeons, and discussing these challenging cases with specialist radiologists will enable optimal management of THR complications.

## Introduction

Total hip replacements (THR) have been performed in the UK from the 1960s and since then we have seen surgical techniques, the design of implants, and imaging modalities rapidly develop (1). The THR is one of the most common and successful procedures performed as a treatment for osteoarthritis (2). As our population continues to age, we will see the incidence of this procedure increase and around 100,000 THRs are already being performed annually with a year on year rise. Around 5–6% of these THRs will undergo revision due to various complications (3). These complications may occur as a result of adverse events that occur at the time of surgery or within the post-operative period (4).

Imaging is an essential component in the work up of patients with THR. Although imaging is not performed by orthopedic surgeons, an understanding of the radiological appearances of these complications, along with the advantages and disadvantages of each modality, will ensure optimal patient care. This paper reviews the different complications and imaging appearances that help evaluate each problem.

## Overview of Imaging Modalities

Evaluation of the THR in the post-operative period relies heavily on imaging together with clinical assessment. Abnormalities detected through imaging should therefore be correlated with the patient's history and examination findings before reaching a diagnosis. Prostheses should be assessed for signs of failure, loosening or migration in the immediate post-operative period with the use of imaging. This imaging provides a baseline for future evaluation of the joint (5). The British Orthopedic Association blue book on good practice relating to THR recommends “radiographic follow-up in the form of AP and lateral X-rays at 1 year, 5 years, and each subsequent 5 years following surgery” (6).

### Plain Radiographs

A plain radiograph or X-ray should be the initial imaging to be performed for any patient with a THR (7). Immediate post-operative radiographs taken whilst the patient is in recovery are ineffective for screening and unsuitable as a baseline for future evaluations (8). Therefore, it is recommended that a departmental standing anteroposterior (AP) pelvic radiograph is performed once the patient is safe to stand. The hips should be in extension and 15 degrees of internal rotation with the center of the X-ray beam focused on the pubic symphysis to ensure the inclusion of the entire hip prosthesis (1).

Information from the initial post-operative radiograph includes an assessment of the quality of the prosthesis and how successful it is likely to be. This imaging is used to assess leg length along with the positioning and fixation of the prosthesis, as well as ensuring the pre-operative surgical aims were achieved (1). Radiographs are also useful for follow up to assess for post-operative complications. These complications can be classified according to their radiological appearance: (i) peri-prosthetic lucencies that could indicate aseptic loosening, infection, or deposition of metallic wear in peri-prosthetic tissues (ii) sclerosis and bone proliferation (iii) component failure/fracture that can be seen in cases of linear wear, dislocation and peri-prosthetic fractures (1, 2, 9, 10). Serial radiographs are the most effective method of detecting prosthesis loosening. The initial post-operative radiographs are important as they act as a reference point for future comparison. For the most useful comparison these serial radiographs should be taken in a consistent manner with regards to the quality of the imaging (11). Serial radiographs offer an assessment of the prosthesis over time and therefore allow the detection of subtle changes. The key role of radiographs in the detection of prosthesis loosening will be discussed further in section Aseptic Loosening and Osteolysis (12).

Although radiographs are able to detect a majority of complications, they have limitations and other forms of imaging are used to detect any pathology that could be missed.

### Computed Tomography (CT)

**Figure 1:**
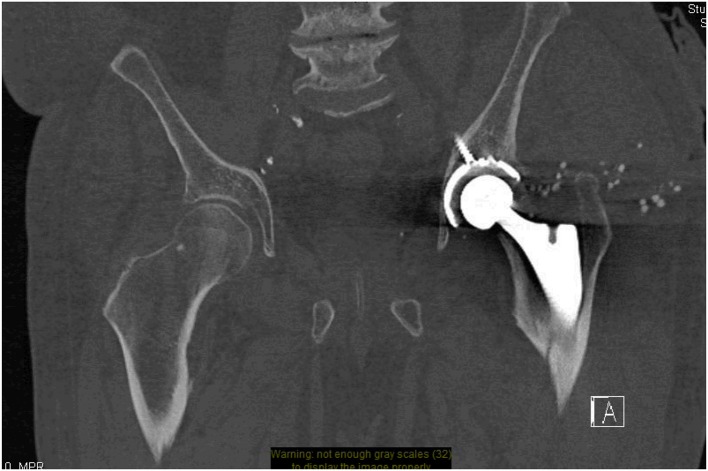
CT pelvis- minimally displaced left proximal femur peri-prosthetic fracture. Small round stimulant beads visible around the left side tracking down toward the left hip wound.

Common indications for CT of a hip prosthesis include normal or unclear radiographic findings when evaluating a painful hip and the evaluation of joint masses, collections around the joint, and ossifications surrounding the soft tissue (13). CT is relatively inexpensive, readily available and easy to perform making it an excellent tool to supplement radiographs when trying to evaluate hip prostheses [Figure 1; (14)]. The use of CT in conjunction with serial radiographs for the early detection of aseptic loosening will be discussed further in section Aseptic Loosening and Osteolysis.

Although CT has been used to investigate metal implants, until recently it has not performed well-due to metal artifact. The intensity of metal artifact depends on the metal used, with most intense artifacts observed with chromium-cobalt implants and less intense artifacts observed with titanium implants (15). The development of technologies such as dual-energy acquisition, adaptive iterative reconstruction and metal artifact reduction (MAR) algorithms have reduced metal artifacts, and improved the quality of CT images (16, 17). This has also reduced the radiation dose that patients are exposed to. New MAR algorithms are constantly being developed and they all vary according to the manufacturer and metal being imaged. It is important to note that new metal artifacts have been reported with the use of MAR algorithms, hence images should be reconstructed with and without these techniques for comparison (18, 19).

Iterative reconstruction results in less image noise, reduces X-ray beam hardening artifacts, diminishes metal artifacts and as a result has become a reconstruction method used for CT (20). Dual energy CT proves efficient for reducing artifact but, due to an insufficient number of photons reaching the detector, is ineffective for imaging hip prostheses. Even when combining this technique with MAR algorithms, it is not superior to the use of these algorithms with conventional CT (21). That being said, a study done by Kasparek et al. (22) concluded that ceramic and titanium knee replacement prosthesis imaged utilizing the “dual-energy CT protocols with mono-energetic imaging provided a significantly better quality image, as well as fewer artifacts, with lower radiation dose compared to single-energy CT.” However, when these protocols are applied to dense metal it is better to perform conventional CT with use of a MAR algorithm (21, 22).

**Figure 2:**
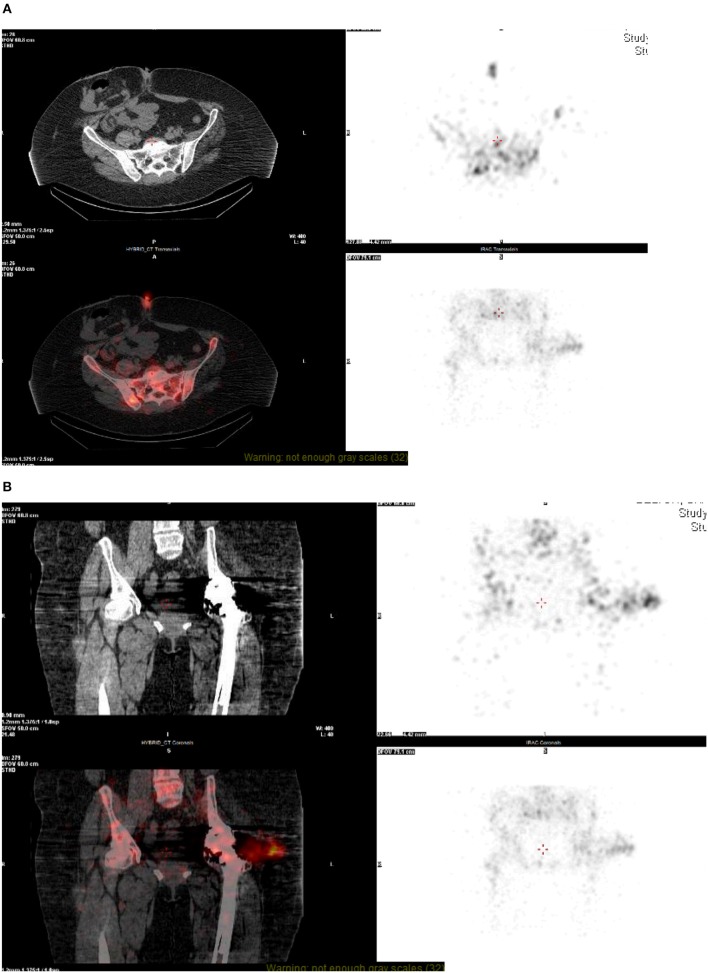
**(A,B)** NM technetium and white cell scan—SPECT CT. On SPECT-CT, there is image degradation seen at the level of the hip joint, despite this, there is some fluid visible lateral to the hip joint which co-localizes to the intense focus of white cell uptake. This fluid appears to track from the hip joint into the deep subcutaneous tissue, representing bursitis, or soft-tissue abscess formation. A focus of uptake can also be seen at the skin surface at the level of the umbilicus, most likely related to recent surgery.

Single photon emission computed tomography with CT (SPECT-CT) is an emerging diagnostic modality that combines the sensitivity offered by bone scintigraphy with the high specificity of CT. SPECT imaging has the advantage of showing the bone's metabolic activity that surrounds the prosthesis and is less prone to be affected by metal artifact compared to MRI. This has the added advantage of more anatomical detail within the images due to the use of CT along with scintigraphy in combination rather than a single modality used in isolation (Figures 2A,B). This modality can be used in the detection of THR complications such as aseptic loosening, peri-prosthetic infection and fractures and heterotopic ossification (23). However, there is still limited literature on its efficacy and clinical validity, and hence it is used where conventional investigations are inconclusive (23, 24).

### Magnetic Resonance Imaging (MRI)

**Figure 3:**
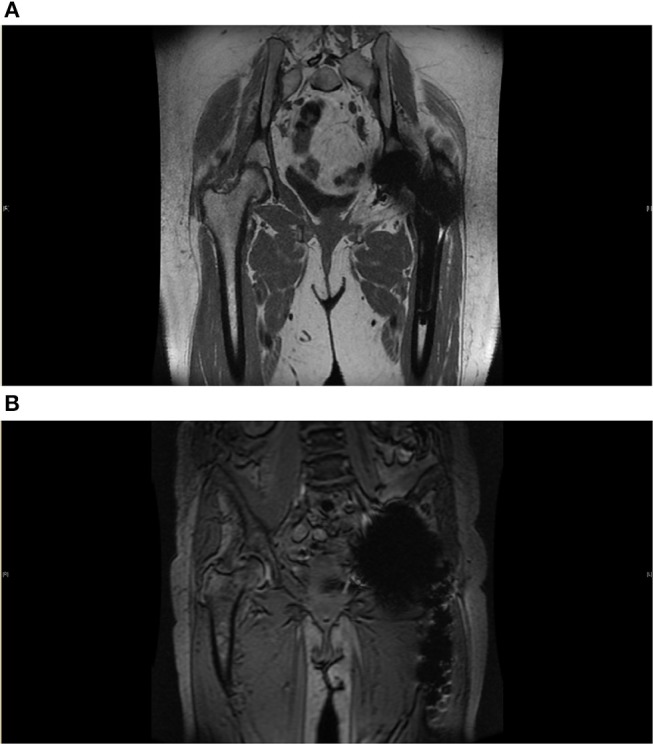
**(A)** Metal Artifact Reduction MRI image. Reduction sequence is utilized here in assessment of prosthesis failure in the left side following THR. Some metal artifact remains. There is no significant joint effusion, soft tissue oedema or collection seen. Common findings following THR can be seen here, this includes fatty atrophy in the short external rotators and adductor brevis. **(B)** Metal artifact reduction MRI image. Here, reduction sequence is utilized in order to minimize aberrations from acting as noise in the assessment of the contralateral joint. There is moderate degenerative change in the superior aspect of the right hip with near complete loss of articular cartilage and bone marrow oedema consistent with osteoarthritis. There is no hip effusion or synovitis.

Magnetic resonance imaging (MRI) has evolved to become an important diagnostic tool for the evaluation of THR in the post-operative period. Optimized pulse sequences and metal artifact reduction techniques have made MRI a useful tool in the diagnosis of soft tissue abnormalities. [Figures 3A,B; (25, 26)]. Metal artifact reduction sequence (MARS)-MRI is commonly used as an umbrella term to describe concepts of metal artifact minimization without compromising image quality. In recent years, several dedicated MRI 3D multispectral imaging sequences have been generated to address metal-related artifact including the multi-acquisition variable-resonance image combination (MAVRIC) and section encoding for metal artifact correction (SEMAC) techniques and their hybrid techniques (26). MAVRIC and SEMAC are fast spin echo based sequences that help to minimize metal artifact (27).

The warp MR sequence combines slice-encoding metal artifact correction (SEMAC) and view-angle tilting (VAT), and aims to achieve less artifact and better image quality for both short tau inversion-recovery (STIR) and T1-weighted sequences. A study in 2018 showed that “compared with turbo spin echo (TSE) MR imaging, SEMAC VAT MR images significantly reduced metal artifact and successfully detected pathological findings otherwise missed by simple radiographs” (26, 27). MAVRIC and SEMAC are the two most widely used techniques for reducing metal artifact and studies have shown that they have similar efficacy; the choice depends on the prosthesis material and the imaging time (26). These artifact reduction techniques have the disadvantage of longer scanning time that limit their use in in less cooperative patients e.g., the elderly or young children (28, 29).

Hybrid metal reduction techniques e.g., SEMAC included VAT for in-plane artifact reduction, have been developed by combining different methods and have started being used in clinical practice. The SEMAC approach has also been extended to a 3D fast spin echo sequence known as sampling perfection with application optimized contrasts by using different flip angle evolutions (SPACE). This allows for “isotropic 3D imaging”; the term isotropic means that “the voxels generated by the 3D acquisition measure the same in each direction, allowing the images to be reformatted with equal resolution in any direction.” A study by Ai et al. in concluded that SEMAC-VAT (2D) and SPACE (3D) revealed a reduction of metal artifacts for different metal implants and high image quality (30). This technique has not however yet been applied to a study involving THRs.

MAVRIC-Selective (MAVRIC-SL) combines the spectral properties of MAVRIC and z-selectivity with VAT principle of SEMAC to improve visualization of THRs. A study done by Choi et al examining MAR with MAVRIC SL in patients with THRs concluded that “MAVRIC SL can significantly reduce metal artifact on 3-T MRI compared with 2D FSE” improving anatomical detail on the images and the diagnostic value of the scans. This modality can also help improve the detection of prosthesis related complications (31).

Ultrashort TE (UTE) is an alternative to the spin echo methods that can be used on tissues with a short T2 time. This can help when evaluating hip prosthesis but no direct comparison has been done between UTE and the spin echo sequences. UTE-MAVRIC is a hybrid technique that benefits from combining UTE that allows for the imaging of short T2 tissues such as cortical bone with MAVRIC that reduces metal artifact. Again, there are limited studies on these methods as they are in their early stages of development (27).

MR imaging in particular plays a vital role in the diagnosis and grading of adverse local tissue reaction as radiography and CT have poor sensitivity due to a poor correlation between symptoms and ion levels (25). This will be discussed later on in section Soft Tissue Abnormalities.

### Nuclear Medicine

**Figure 4:**
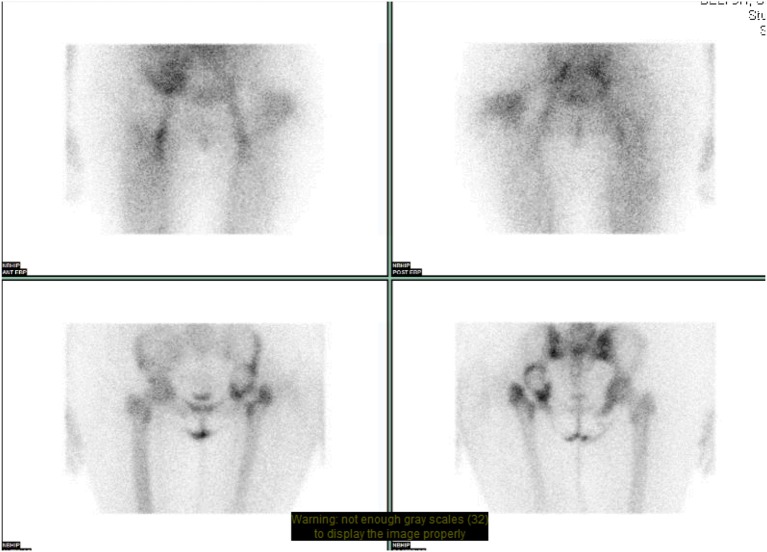
NM dual phase bone scan pelvis. Increased blood pooling lateral to proximal left femur. Increased uptake approximates to acetabular cup and greater trochanter. Suggests loosening of left sided acetabular component with infection involving the left greater trochanter.

Bone scintigraphy has been used early on to identify prosthetic failure in THR. Technetium-99m (^99m^Tc) labeled diphosphonates, usually methylene diphosphonate (MDP), are used for this study. There is mixed evidence regarding the specificity and sensitivity of the study. Although it is a sensitive indicator of a failed prosthesis, it cannot reliably identify the exact cause of failure (5). At present, this test can be used as a screening tool where a normal study makes it unlikely that a patient's symptoms are related to the prosthesis (32). CT and MRI are accurate in identifying the diagnosis of most causes of THR complications except infection (Figure 4). Over the years, techniques have been developed to overcome the limitations of bone scintigraphy. Gallium scintigraphy was an early technique where increased uptake in infection would be observed secondary to uptake of gallium by bacteria and increased blood flow to sites with infection or inflammation. Research evaluating the success of these studies showed a mixed picture, but in most series Gallium scintigraphy was not accurate or sensitive enough to reliably diagnose peri-prosthetic infection and only offered a modest improvement when compared to bone scintigraphy alone (33).

A significant development to improve imaging for infected prosthesis was leukocyte labeled scintigraphy that allows the labeling of cells with radioactive substances that then migrate to the area of infection (34). Most of the leukocytes labeled are neutrophils hence the procedure detects bacterial infections that are neutrophil mediated. The procedure is less useful for illnesses such as most opportunistic infections and spinal osteomyelitis (35). Leukocyte labeled imaging theoretically should be a well-suited modality of imaging since white cells are unlikely to accumulate at sites of increase bone turnover unless there is an infection present. Most studies however reported that the test, although specific for joint infection, had poor sensitivity. The poor sensitivity is believed to be due to the long term nature of the process; by the time the patient would have imaging performed, the infection would have ceased or is resolving and would not be detected due to the lack of neutrophilic response. Some studies found the test to be sensitive but not specific, probably due to joint inflammation not necessarily due to infection (36).

Another problem with leukocyte labeled scintigraphy relates to the interpretation of the images. Interpreting images involves comparing the activity in the region of interest with the activity at a region presumed to be normal. Leukocyte labeled studies are interpreted as positive for infection when uptake in the region of interest is more than other “unaffected” areas. The intensity of uptake in an area of presumed infection and an area presumed normal is however variable (37).

Currently the labeled leukocytes that are used, collect in the bone marrow as a result of phagocytosis by the reticuloendothelial cells in the marrow. The distribution of the bone marrow closely parallels that of the region of hematopoiesis in most conditions (38, 39). Leukocyte-marrow scintigraphy allows for this problem to be solved as it allows for assessment of the bone marrow as well as areas of infection. Therefore, only studies that demonstrate activity on the leukocyte image without corresponding activity on the marrow image should be interpreted as positive for infection (37). Love et al. (40) reported on 150 failed joint prostheses with confirmed final diagnoses. In this investigation, “the sensitivity, specificity, and accuracy of leukocyte-marrow scintigraphy were 96, 87, and 91%, respectively. The test was significantly more accurate than bone (50%), bone/gallium (66%), and leukocyte/bone scintigraphy (70%) in their population.” These results confirm that leukocyte-marrow scintigraphy is the modality of choice for accurately diagnosing prosthetic joint infection and reassures us of its superiority over other nuclear medicine imaging (40).

Despite the diagnostic accuracy, leukocyte-marrow scintigraphy is time consuming and labor intensive as well as being limited in availability. Due to the handling of blood products it can also be deemed to be potentially hazardous. ^18^F-flouro-2-deoxyglucose positron emission tomography (FDG-PET) enables visualization of inflammatory cells during infection. This may be a suitable alternative to combined leukocyte-marrow scintigraphy as it only requires one injection and is more widely available. Published results to date however are inconclusive with contradictory findings. Due to the lack of evidence in favor of FDG-PET scanning, leukocyte-marrow scintigraphy is still more extensively used (39).

F18-fluoride-PET (fluoride-PET) bone imaging is an evolving technique that has demonstrated good results in a few studies. Kumar et al found that dual phase 18F fluoride PET/CT has considerable potential in differentiating septic and aseptic loosening of hip prostheses. In another study, Ullmark et al. studied bone mineralization around the femoral component of cementless THRs and concluded that fluoride-PET was a useful for analyzing bone mineralization around hip prostheses (39). In 2011, Naomi Kobayashi et al. published a prospective study of ^18^F-NaF PET in 65 hip prostheses. This test had a “sensitivity and specificity of 81 and 80%, respectively, and for aseptic loosening, this test yields a sensitivity and specificity of 95 and 82%, respectively” (41, 42). Another study by Kobayashi et al in 2016 showed F-Fluoride PET was able to identify the “accelerated local bone turnover” in patients with a painful THR (43). More recently, van der Vos et al. showed the first clinical results of a newly available MAR tool for PET/CT used on 21 patients. This new algorithm was able to provide added confidence in image interpretation and a positive impact of quantitative accuracy of PET/CT when used with metal prostheses (44).

Although few studies have shown the usefulness of this modality they have shown positive results, and leukocyte- marrow scintigraphy is currently the most accurate nuclear medicine choice to diagnose infection.

### Ultrasound (US)

**Figure 5:**
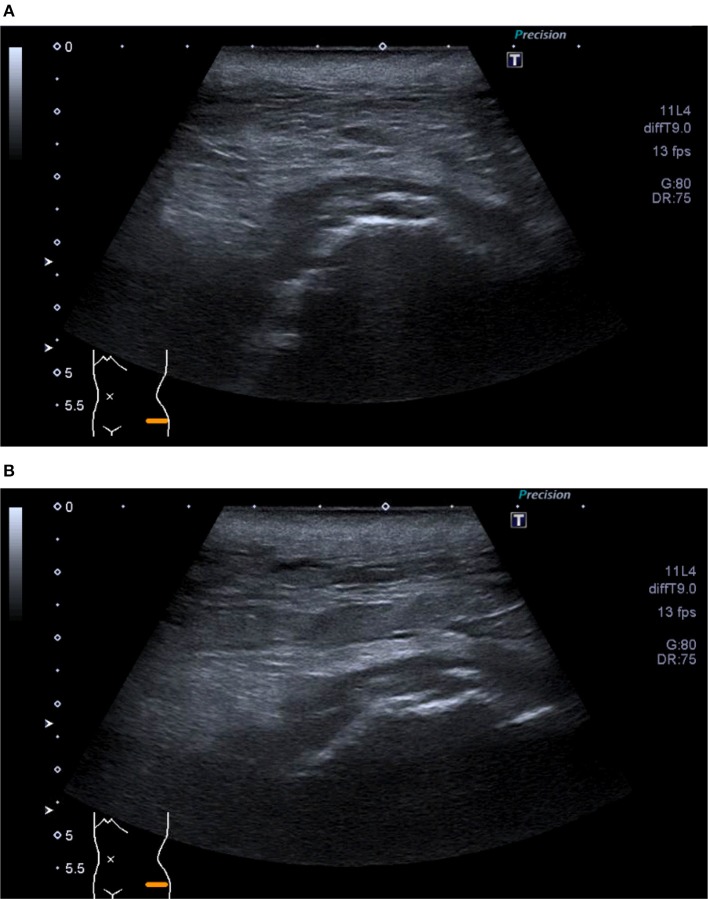
**(A,B)** Ultrasound of left hip—Superficial collection at site of wound tracking down to deep hip joint surface. Extension to borders of skin markers.

Ultrasound (US) is not recommended as the first line examination to assess peri-prosthetic bone complications due to the deep location of the hip prosthesis and the inability of sound to penetrate bone or metal (45). Ultrasound however still has a role in identifying peri-prosthetic fluid collections and the presence of soft tissues sinus tracts (Figures 5A,B). It also has the added benefit of allowing hands-on contact with the patient that can help localize pain or tenderness to an anatomical location. Ultrasound can also be used for guided fluid aspirations to detect infection and to visualize the surrounding soft tissues and bursae (46).

## Complications

Complications of THR which commonly lead to revision surgery include aseptic loosening and osteolysis, instability, dislocation, infection, peri-prosthetic fractures, soft tissue abnormalities, component failure, and heterotopic ossification. Here, we highlight the different imaging modalities available for diagnosing each complication.

### Aseptic Loosening and Osteolysis

Aseptic loosening remains the most significant long term complications following THR (47). Aseptic loosening is described as a loss of fixation of the implant which can occur as a result of “inadequate initial fixation, mechanical loss of fixation over time, or biologic loss of fixation caused by particle-induced osteolysis around the implant” (48). Although there is no agreed definition of aseptic loosening, the formation of a “synovial-like membrane” between the prosthesis and the bone is frequently described (49). The prosthesis can then separate from the host bone resulting in mechanical loosening (50, 51). This process can eventually cause the migration of either the femoral or acetabular component from their original position which can be detected on imaging (47).

Serial radiographs, as mentioned earlier, offer the most value when trying to assess for prosthesis loosening. A zone of lucency measuring <2 mm with either a cemented or non-cemented prosthesis could represent a stable reaction to the cement or fibrous bony ingrowth, respectively (1). Lucency of more than 2 mm at the cement-bone or metal-bone interface around the entire circumference would indicate loosening of the prosthesis (52). Progressive lucent lines around the prosthesis on serial imaging is highly suggestive of loosening as well (53).

For the interpretation of plain radiographs, radiological criteria are used to assess loosening of both cemented and uncemented THRs. In a review of 171 cemented THRs by Harris et al. (51), the likelihood of loosening was defined as “possible,” “probable,” or “definite” based on defined radiographic criteria. The presence of a radiolucent line of 50–100% of the cement-bone border is interpreted as “possibly loose” while the radiolucency found in 100% of the interface is referred to as “probably loose.” Migration of the component or a cement mantle fracture would indicate a “definitely loose” component (14, 51). The more progressive the lucent radiological lines are, the more likely prosthesis loosening is present. For cemented THRs, further radiographic signs that may indicate loosening described by Engh et al. include migration as previously mentioned and reactive lines seen as endosteal densifications parallel to the lining of the prosthesis.

The expected appearance of cementless prostheses is more varied. On the basis of studies of an asymptomatic population, the following appearances are accepted as normal. In stable non-cemented prostheses lucent zones at the metal-bone interface do occur, as it usually is a combination of bone ingrowth and fibrous tissue ingrowth that provides the fixation in most cases. This fibrous tissue presents as a lucent zone at the interface. Again, it should be stable and within a range of 1–2 mm. Cortical thickening can be seen in normal uncemented prosthesis as well as stress shielding in areas that are relatively unstressed. The forces are transmitted through the femoral stem and stress shielding is seen as osteoporosis in the proximal femur with thinning of the cortex and bone resorption of the femoral neck. This is seen laterally at the greater trochanter and medially as calcar resorption or otherwise known as calcar round-off (54, 55). Other observed features include pedestal formation (endosteal densifications at the tip of the stem), calcar hypertrophy, and “spot welds” (endosteal new bone formation bridging from the cortical bone to the prosthesis) (54).

The most reliable radiographic signs of loosening in cementless hip prosthesis are progressive subsidence (>10 mm), migration of the acetabular cup, or varus tilt of the femoral stem, altogether known as “component migration.” These abnormalities may be very subtle often without developing a radiolucent appearance. Hence, serial radiographs are important for diagnosis (55). Measurements to evaluate acetabular loosening include superior migration (top of cup to transischial tuberosity line), medial migration (medial cup to teardrop or another medial landmark), and lateral inclination (judged by measuring the angle of the cup relative to the transischial tuberosity line) (55).

CT can be utilized if the radiograph appears normal or displays subtle areas of lucency which can be difficult to interpret. Radiolucent areas can, when stable, be clinically insignificant however these findings need to be closely monitored as they can indicate probable loosening (13). CT imaging findings of <2 mm radiolucent areas around components or between a cement mantle and bone are considered normal radiographic findings. These lines could however indicate loosening in a symptomatic patient. Areas >2 mm may be considered normal if no changes are seen on serial imaging (13).

SPECT-CT is able to provide further information to diagnose aseptic loosening of a prosthesis which otherwise would not be available when using plain radiography or CT alone. Aseptic loosening on SPECT-CT would commonly present as focal areas of lucency around the prosthesis which are >2 mm with corresponding increased uptake on SPECT. An increase in uptake on serial SPECT images would also suggest loosening (23).

Plain radiographs have the highest diagnostic accuracy in the evaluation of aseptic loosening of the acetabular component. With subtle or unequivocal radiograph findings, the use of CT and SPECT-CT can be useful to aid diagnosis of this complication. Serial imaging with radiographs or CT should be used to assess for component migration when the degree of loosening is unclear.

### Instability and Dislocation

Dislocation is the second commonest reason for revision of a THR (56). More than 75% of all dislocations occur within the first year following surgery (56). The risk of dislocation is influenced by patient factors such as age, sex, previous surgery as well as surgical factors such as surgical approach, prosthesis choice and surgeon technique (29). Most dislocations following a THR are isolated events and hence can be managed non-operatively. Some patients may require a revision to prevent recurrent instability (57, 58).

Radiographic evaluation with two views is sufficient to diagnose a dislocation. Normal findings on the post-operative radiograph include: acetabular lateral inclination angle of 30-50 degrees or acetabular anteversion angle 5–25° (10). The femoral head and femoral stem should both be well-centered (10).

It is important to note that there are several radiological methods for measuring the anteversion of the acetabular component after THR, and a single standardized method has not been established. The definition of anteversion also differs and Murray et al. described three types of anteversion of the acetabular component: operative seen intra-operatively, radiographic seen on post-operative radiographs, and anatomical seen on CT (59). Research conducted by Park et al. (60) looked at the anteversion of the acetabular component measured on the AP radiograph of the pelvis using six different methods. They found that the Liaw et al. method was the most accurate and the Woo and Morrey method showed the highest intra- and inter-observer reliability (60).

CT can be used to provide a more detailed assessment of the degree of anteversion when this is unclear on plain radiographs. These images can also be used to plan revision surgery if this is required (10).

For the diagnosis of a dislocated hip replacement, plain radiographs are used for the vast majority of cases and should be the modality of choice when suspecting this complication.

### Infection

The clinical and imaging diagnosis of infection in a THR is challenging (25). It is the third commonest reason for revision and occurs at a rate of 1–2% in primary THR (50). The initial work up of these patients should include a detailed history, examination and blood tests including CRP and WCC to detect infection as well as cultures to isolate a causative organism (61). With regards to imaging, there is no defined gold standard technique to diagnose infection. Radiographs form part of the initial work up however these images may be entirely normal in cases of acute infection. In chronic infection, plain radiographs may show “wide irregular radiolucency around the cement–bone or metal–bone interfaces and/or bone destruction” that would indicate osteolysis around the prosthesis. A single radiograph however would not be sufficient to differentiate between infectious and non-infectious causes and the use of additional imaging techniques becomes essential (10).

With improvements in reducing beam hardening artifact, CT has become a useful modality in these scenarios as it enables the analysis of peri-prosthetic bone as well as soft tissue. Radiological changes which indicate aseptic loosening such as lucent lines and areas of osteolysis are also suggestive of infection. Analysis of the soft tissues is essential and this aids the clinician to differentiate between aseptic loosening and infection (61). Intravenous contrast used with CT helps visualize soft tissue and fluid collections, or enhances the contours of an inflammatory synovium. Soft tissue findings are accurate when detecting infection with 100% sensitivity and 87% specificity. Fluid collections have a 100% positive predictive value, therefore using the best modality to detect these is key (61).

Ultrasound is less contributory for imaging of the hip due to the depth of the prosthesis but can be used to analyse soft tissues. It is a sensitive modality for evaluating a hip joint effusion which can be seen as hyperechoic fluid displacing the joint pseudocapsule. Using ultrasound can also be done prior to hip aspiration to ensure accurate retrieval of any joint fluid which can provide pain relief to the patient and important diagnostic information (46). This modality is an excellent way to evaluate any superficial soft tissue collections without metal artifact obscuring the imaging. Soft tissue thickening and hyperemia seen on Doppler sonograms may suggest the presence of infection (46).

Over the years, different techniques have been developed within nuclear medicine to improve the accuracy in detecting joint infections. Bone scintigraphy alone has limited use due to its poor specificity in comparison to other imaging modalities which are available. Therefore, hybrid techniques have been developed to overcome these limitations (61). Gallium and leukocyte labeled scintigraphy was used to improve diagnostic accuracy for infection, but this has now been replaced in most circumstances by leukocyte-marrow scintigraphy (61). Labeled leukocytes combined with sulfur colloid bone marrow scintigraphy remains the modality of choice for the diagnosis of prosthetic joint infection (52). As previously discussed in section Nuclear Medicine, the results for leukocyte-marrow scintigraphy in comparison to leukocyte scintigraphy alone report an accuracy rate of 90% and therefore is the best modality to identify this complication (61).

Due to the limited availability and increased costs, leukocyte-marrow scintigraphy may be challenging to perform therefore CT and SPECT-CT would be a more preferred modality to use when initially suspecting prosthesis infection. If the results from these modalities are inconclusive then it would be advised to proceed with techniques such as leukocyte-marrow scintigraphy to ensure a diagnosis is made (7).

### Peri-Prosthetic Fracture

Peri-prosthetic fractures are becoming increasing common in the setting of THR due to the increasing numbers of primary and revision THRs being performed (62). Prevalence of this complication is around 4% in primary THRs with rates in revision procedures being higher (62). These fractures occur more often in the femoral component rather than the acetabulum and can occur either intra-operatively or post-operatively (10). It is important to identify these fractures early as delay to surgery has been identified as a strong negative prognostic indicator for subsequent THR revision (63). Diagnosis is usually made with a clinical history of pain and injury along with imaging to confirm this. The Vancouver classification is used to divide these fractures according to location, quality of bone stock and stability of stem. This classification system allows a surgeon to differentiate stable prostheses from unstable prostheses, and decide on the need for osteosynthesis or revision (64).

Radiographs are key in the initial work-up for anyone with a suspected peri-prosthetic fracture and should be sufficient for diagnosis in a majority of cases (65). Anteroposterior radiographs of the pelvis and hips, and lateral radiographs of the affected hip should be obtained initially. These should then be compared with previous radiographs to detect a fracture. Radiographs can have a low sensitivity in cases where the fractures are subtle and this would necessitate the need for further imaging (11).

CT imaging can be used when radiographs are negative or when a fracture is subtle around the acetabular component. These images can also provide information on possible loosening of the prosthesis and help decide whether further investigations are required to rule out possible joint infection (66). CT can also be helpful to aid surgical planning especially in cases where fractures are more complex (10).

SPECT-CT can be used in a select number of cases where the SPECT component is able to identify evidence of delayed union or healing when there is a known peri-prosthetic fracture (10). Pseudoarthosis of the greater trochanter is an isolated fracture of the greater trochanter. This occurs most commonly following a trochanteric osteotomy which may be used in THR when there is severe femoral deformity. SPECT-CT is especially useful in these cases as it can accurately identity non-union of the greater trochanter which may otherwise not be clear on CT alone (24).

MRI can be used for cases where the fractures may be associated with osteolysis or where the fractures are subtle on the CT. However, due to the susceptibility of this imaging to metal artifact these fractures may be difficult to appreciate even on MRI therefore there is limited use of this modality when this complication is being suspected (10).

### Soft Tissue Abnormalities

Soft tissue abnormalities such as greater trochanteric bursitis and other fluid collections, tendinopathies including iliopsoas impingement as well as adverse local tissue reactions (ATLR) secondary to metal on metal (MOM) THR implants can present as a painful hip with normal radiographic findings (52).

Greater trochanteric bursitis can occur following a THR however is relatively minor and easily treatable in comparison to other complications mentioned. This can present as pain and tenderness at the lateral aspect of the hip and as well as this presentation being attributed to bursitis it can also occur as a result of tears or tendinosis of the gluteal muscles (11). MARS MRI sequences such as MAVRIC and SEMAC mentioned in section Magnetic Resonance Imaging (MRI) are the preferred imaging modality to determine the cause of trochanteric pain syndrome and solves the issue of metal artifact obscuring the soft tissues. Ultrasound can also be used for these complications as it allows us to visualize any fluid collections and US guided injections can provide symptomatic relief to the patient (11).

Impingement of the iliopsoas tendon is also a potential cause for a persistently painful hip following THR and has a prevalence of 4%. In most cases this occurs as a result of friction between the tendon and an oversized or mal-positioned acetabular cup (10). Ultrasound is often used as the preferred imaging modality when suspecting tendon pathology as it allows for dynamic assessment of the joint and the use of an US-guided steroid injection could provide short-term symptomatic relief. Ultrasound also has the added benefit of being readily available and avoids excessive radiation exposure (46).

An important soft tissue abnormality to identify is adverse soft tissue reactions (ATLR) which can occur as a result of Metal on metal (MOM) THRs. ALTRs are caused by an inflammatory response to the small metal debris created by the MOM bearing surface. This inflammatory response can lead to “metallosis, formation of a bursal soft tissue growth known as a pseudotumor, and generalized synovitis and tissue damage.” These reactions can subsequently cause destruction of the surrounding soft tissue, joint and capsule therefore early diagnosis is essential to allow for timely revision surgery (67).

Imaging is key to diagnose ALTR as research reports a poor correlation between presenting symptoms and the measurement of ion levels in the blood. Metal artifact reduction sequence (MARS) MRI is the main imaging modality used in suspected ALTR and is superior at identifying soft tissue damage in comparison with other imaging modalities (25). MARS MRI enables us to visualize dehiscence of the capsule, fluid collections around the prosthesis, osteolysis, and pseudotumours all occurring as a result of ALTR (68). Pseudotumors on MRI can range from thin-walled cysts to solid masses and can be associated with synovitis, fluid collections, and metal debris (25).

MARS MRI can also provide a detailed assessment of the degree of soft tissue destruction and with this information we are able to grade the severity of the reaction which can have an implication on management (68).

In the presence of normal radiological findings, MARS MRI should be used as a key imaging modality to allow for the identification of any soft tissue abnormalities. In cases where a fluid collection is suspected ultrasound should be considered as it would allow us to simultaneously aspirate the joint if this is required.

### Component Failure

Component failure can affect both the femoral and acetabular components and will depend on the type of prosthesis being used. Stress or metal fatigue of the femoral stem can cause it to break usually because the stem is less yielding than the surrounding bone and this would be evident in most cases on plain radiographs (52). Asymmetrical positioning of the femoral head within the acetabular cup seen on a plain radiograph is a definite sign of liner wear which can typically occur in a replacement where there is a polythene component. Serial radiographs may be necessary to detect any subtle changes secondary to liner wear (10). Alternatively, the polyethylene liner around the acetabulum can break off and completely detach from the outer metal cup (52). Plain radiograph would show the metal femoral head superiorly located in the acetabular cup with possible displaced pieces of the polythene liner or metal debris visible around the prosthesis (52). Ceramic on ceramic prostheses can be prone to fractures due to its brittleness. If these fractures are displaced, they can be detected on plain radiographs as areas of irregularity. Some fractures may not be seen on plain radiograph and therefore CT would be more useful in these cases (10, 13).

### Heterotopic Ossification

Heterotopic ossification (HO) is the “abnormal formation of mature lamellar bone within extra-skeletal soft tissues.” HO after THR can cause pain, decreased range of motion and impingement, and can have an impact on functional outcome (69).

Heterotopic ossification (HO) is the abnormal growth of mature lamellar bone in non-skeletal tissues including muscles, tendons and other soft tissue and can occur following THR. It is estimated that around 8% of THR patients experience pain as a result of heterotopic ossification however around 90% of patients will have radiographic evidence of this process. On post-operative radiographs this process can be seen as small clinically insignificant foci and is usually asymptomatic. In a small number of patients, large areas of HO can result in significant impingement of the adjacent soft tissues causing disabling pain for the patient (10, 70). This would require excision of the affected areas and imaging is required for the accurate diagnosis of this process. Plain radiographs are appropriate for initial assessment and the severity of the heterotopic bone can be classified according to several systems using this modality. One system traditionally used is the Brooker classification which divides severity into 4 types ranging from Class I which is described as islands of bone in the soft tissue to Class IV which is described as apparent ankylosis of the hip joint. Ossification typically appears on radiographs as areas of hyperdensity and this gradually matures to solid bone by 3 months. CT will mirror the findings on plain radiographs but when compared with each other, CT is able to identify a soft tissue masses much earlier enabling a prompter diagnosis (70). CT is also more useful to identify the exact anatomical location of the ossification and is used for surgical planning. The different stages of ossifications also appear to be clearer on CT imaging which can give us more information about the severity and how necessary surgical excision is Amar et al. (70).

## Discussion

A wide range of complications may be encountered following THR. These include: aseptic loosening and osteolysis, instability, dislocation, infection, peri-prosthetic fractures, soft tissue abnormalities, component failure, and heterotopic ossification. When assessing symptomatic patients, it is important to correlate the clinical and radiological findings. With regards to imaging, plain radiographs would remain the initial investigation to be performed with patients who have undergone THR who present with pain. The initial post-operative radiographs are important as they act as a reference point for future comparison. Serial radiographs can help to identify any subtle changes and are most useful when trying to identify signs of aseptic loosening. Instability and dislocation, peri-prosthetic fractures, component failure and heterotopic ossification can also be easily diagnosed using plain radiographs. Nevertheless, radiographs have limitations and if the diagnosis is not clear, it will be necessary to proceed with further imaging to diagnose these particular complications. Based on the review of the literature available, CT would be an appropriate modality to use in these scenarios due to its widespread availability, cost effectiveness and accuracy in being able to diagnosis complications that may have been missed on radiographs. In cases where infection is suspected SPECT-CT is a suitable option as it is easily available when compared to other nuclear medicine imaging. In centers where nuclear medicine is readily available, leukocyte-marrow scintigraphy remains the most accurate modality to diagnose infection in patients with a THR. For soft tissue abnormalities, the use of MARS MRI is especially useful as it enables us to clearly visualize soft tissue. The recent development of MARS means that the THR does not obscure the images allowing us to make an accurate diagnosis. MARS MRI is of particular use in MOM prosthesis where ALTR is suspected. To aid the diagnosis of fluid collections, ultrasound can be useful as it provides a dynamic examination whilst allowing the clinician to provide therapeutic benefit in the form of an intra-articular injection or aspiration. However, due to the deep location of the hip prosthesis and the inability of sound to penetrate bone or metal it has a limited role to diagnose other complications and so the use of CT would be preferable.

## Conclusion

Plain radiographs should always be the initial investigation as this modality is able to diagnose the majority of complications. Other modalities such as CT, scintigraphy, ultrasound, and MRI are all important in cases where a plain radiograph appears normal or where changes are subtle and difficult to clearly identify. CT would be the most appropriate modality to use following a radiograph unless the clinician is suspecting a soft tissue abnormality, in which case MARS MRI would be more beneficial. Being aware of the imaging modalities available, and discussing these challenging cases with specialist radiologists should help ensure optimal management.

## Permission to reuse and Copyright

Figures, tables, and images will be published under a Creative Commons CC-BY license and permission must be obtained for use of copyrighted material from other sources (including re-published/adapted/modified/partial figures and images from the internet). It is the responsibility of the authors to acquire the licenses, to follow any citation instructions requested by third-party rights holders, and cover any supplementary charges.

## Author Contributions

NM was involved with writing the manuscript in consultation with WK. KT and CG were involved with acquisition of images and reviewing the final manuscript.

### Conflict of Interest Statement

The authors declare that the research was conducted in the absence of any commercial or financial relationships that could be construed as a potential conflict of interest.
